# The Role of Lymphatic Vascular Function in Metabolic Disorders

**DOI:** 10.3389/fphys.2020.00404

**Published:** 2020-05-05

**Authors:** Pieter R. Norden, Tsutomu Kume

**Affiliations:** Feinberg Cardiovascular and Renal Research Institute, Department of Medicine, Feinberg School of Medicine, Northwestern University, Chicago, IL, United States

**Keywords:** obesity, lymphatic, inflammation, nitrous oxide, vascular endothelial growth factors, transcription factor, metabolic syndrome

## Abstract

In addition to its roles in the maintenance of interstitial fluid homeostasis and immunosurveillance, the lymphatic system has a critical role in regulating transport of dietary lipids to the blood circulation. Recent work within the past two decades has identified an important relationship between lymphatic dysfunction and patients with metabolic disorders, such as obesity and type 2 diabetes, in part characterized by abnormal lipid metabolism and transport. Utilization of several genetic mouse models, as well as non-genetic models of diet-induced obesity and metabolic syndrome, has demonstrated that abnormal lymphangiogenesis and poor collecting vessel function, characterized by impaired contractile ability and perturbed barrier integrity, underlie lymphatic dysfunction relating to obesity, diabetes, and metabolic syndrome. Despite the progress made by these models, the contribution of the lymphatic system to metabolic disorders remains understudied and new insights into molecular signaling mechanisms involved are continuously developing. Here, we review the current knowledge related to molecular mechanisms resulting in impaired lymphatic function within the context of obesity and diabetes. We discuss the role of inflammation, transcription factor signaling, vascular endothelial growth factor-mediated signaling, and nitric oxide signaling contributing to impaired lymphangiogenesis and perturbed lymphatic endothelial cell barrier integrity, valve function, and contractile ability in collecting vessels as well as their viability as therapeutic targets to correct lymphatic dysfunction and improve metabolic syndromes.

## Introduction

The growing epidemic of obesity is characterized predominately by the accumulation of excess adipose tissue, which is due to an array of causes including the transition to an increasingly sedentary lifestyle and diets containing processed foods high in calorie content. The progression of this epidemic presents a major challenge to chronic disease prevention, including predisposal to the development of disability, depression, certain cancers, type 2 diabetes (T2D), metabolic syndrome, and cardiovascular disease (Grundy, 2004; De Pergola and Silvestris, 2013; Hruby and Hu, 2015; O’Neill and O’Driscoll, 2015; Ortega et al., 2016). In the United States, severe estimates predict that 86.3% of adults will be overweight or obese by 2030 (Wang et al., 2008), thus underscoring the critical need for development of novel therapeutic treatments in addition to preventative measures and lifestyle changes. Emerging evidence generated within the past two decades has identified key mechanisms relating lymphatic vascular dysfunction with the pathogenesis of obesity, T2D, abnormal lipid metabolism and metabolic syndrome. In particular, the development and progression of metabolic syndrome, obesity, and T2D in some cases is associated with abnormal lymphangiogenesis and impaired function of collecting vessels during lipid transport and immune surveillance (Harvey, 2008; Dixon, 2010; Scallan et al., 2016; Escobedo and Oliver, 2017; Cifarelli and Eichmann, 2019). This review summarizes our current knowledge of the mechanisms by which lymphatic dysfunction contributes to pathogenesis of obesity, diabetes, and metabolic syndrome; focusing on the role of inflammation, transcription factors, vascular endothelial growth factor (VEGF)-signaling and nitric oxide (NO) signaling. Additionally, we discuss recent therapeutic developments targeting lymphatic function for the treatment of diabetes, obesity, and metabolic syndrome.

## The Lymphatic Vascular System

Unlike the closed blood circulatory system, the lymphatic system consists of a blind-ended, unidirectional network of absorptive vessels and secondary lymphoid organs, which include lymph nodes, spleen, Peyer’s patches, adenoids, and tonsils, in which lymph collected from the interstitial space is eventually drained into the blood vasculature via transport through the thoracic duct into the subclavian vein (Ruddle and Akirav, 2009; Choi et al., 2012). This unidirectional transport mechanism serves to support the critical physiological roles of the lymphatic vasculature in maintaining interstitial fluid and protein homeostasis, developing an immune surveillance response by transporting antigen and antigen-presenting cells to lymph nodes, and facilitating the uptake of vitamins and dietary lipids, transported by lipoprotein particles known as chylomicrons, into the mesenteric lymphatic vessels from the digestive tract via the intestinal lacteals (Schulte-Merker et al., 2011; Randolph et al., 2017; Santambrogio, 2018; Cifarelli and Eichmann, 2019). During lymph formation, interstitial fluid containing water, solutes, and immune cells is initially transported into the lumen of highly permeable lymphatic capillary vessels. These vessels consist of lymphatic endothelial cells (LECs) arranged in an oak-leaf pattern characterized with discontinuous, overlapping, “button-like” cell-cell junctions (Baluk et al., 2007). Hydrostatic pressure gradients acting across the lymphatic wall facilitate the opening or closure of these overlapping cell-cell junctions, thus functioning as a primary valve system (Schmid-Schonbein, 2003). Additionally, capillary LECs are also directly attached to the extracellular matrix (ECM) by anchoring filaments (Leak and Burke, 1968). High interstitial fluid pressure also facilitates the opening of overlapping junctions by stretching these anchoring filaments to promote the transport of interstitial fluid into the capillary lumen. Increased intraluminal pressure then promotes the closure of overlapping junctions to inhibit lymph leakage (Schmid-Schonbein, 2003; Sabine et al., 2016). Lymph formed in the capillaries is then passively transported to collecting vessels where LECs are arranged into contractile segments, known as lymphangions, which are separated by intraluminal, bi-leaflet lymphatic valves. These lymphangion regions are additionally coated by basement membrane and lymphatic muscle cells (Muthuchamy et al., 2003). In contrast to the lymphatic capillaries, LECs in collecting vessels form continuous “zipper-like” cell-cell junctions to prevent lymph-leakage during transport (Sabine et al., 2016). Coordination of several forces then promotes the propulsion of lymph within the collecting vessels and maintenance of unidirectional transport. This includes both intrinsic pump forces, derived from contraction of lymphatic muscle and extrinsic forces generated from surrounding tissues (e.g., respiration and contraction of cardiac and skeletal muscle). Additionally, regulation of the opening and closure of intraluminal valves supports unidirectional transport of lymph and prevents backflow. Increases in luminal lymph pressure within lymphangions drives the closure of valve leaflets as they are pushed together. As lymph pressure continues to increase within the lymphangion, the lymphatic endothelium stretches and activates the lymphatic pump, which opens valves to transport lymph to sequential lymphangions (Zawieja, 2009; Bertram et al., 2011; Sabine et al., 2016).

During murine embryonic development, formation of the early lymphatic vascular system is initiated at embryonic day (E) 9.5 as a subset of endothelial cells in the cardinal vein begin to express the Prospero-related homeobox 1 (PROX1) transcription factor, that acts as a master lymphatic regulator. These cells then differentiate into LECs, and migrate to establish primitive lymphatic vessels, identified as the jugular lymph sac, by E11.5 (Wigle and Oliver, 1999). However, more recent evidence has also demonstrated the contribution of other tissue-associated vascular progenitor cells as well as non-endothelial cells to early lymphatic vasculature morphogenesis and the development of the primitive lymphatic vascular network (Bernier-Latmani et al., 2015; Klotz et al., 2015; Martinez-Corral et al., 2015; Stanczuk et al., 2015; Kazenwadel and Harvey, 2016). Several critical signaling pathways are involved in this process including VEGF-C/-D activation of the VEGFR-3 receptor tyrosine kinase, regulation of key downstream gene expression by SOX18, PROX1, GATA2, and FOXC2 transcription factors, as well as Notch1, FGF2/FGFR1, ANG2/TIE2, and EFNB2/EPHB4 signaling, which have been previously reviewed in great detail in articles we refer to the reader (Tammela and Alitalo, 2010; Schulte-Merker et al., 2011; Yang and Oliver, 2014; Zheng et al., 2014). We discuss the progression of metabolic syndrome derived from lymphatic dysfunction as a result of impaired signaling in several of the pathways mentioned, and in contrast the effect of chronic inflammation derived from obesity and metabolic syndrome on several of these signaling pathways, resulting in lymphatic dysfunction, in greater detail below.

## The Relationship of Metabolic Syndrome, Inflammation, and Lymphatic Dysfunction

Numerous studies have demonstrated a role for obesity in the induction of a chronic low-grade inflammatory response (Lumeng and Saltiel, 2011; Saltiel and Olefsky, 2017). In human obese and T2D patients, evidence has shown that several inflammatory markers are elevated, including white blood cell counts and plasma levels of coagulation factors (PAI-1 and fibrinogen), acute-phase proteins (C-reactive protein and serum amyloid A), pro-inflammatory cytokines (tumor necrosis factor (TNF)-α, interleukin (IL)-1β, and IL-6), as well as proinflammatory chemokines (Pickup et al., 1997; Yudkin et al., 1999; Bastard et al., 2000; Haffner et al., 2005; Bruun et al., 2006; Belalcazar et al., 2013; Esser et al., 2014). Furthermore, in most cases increases in these pro-inflammatory markers also positively correlated with insulin resistance and metabolic syndrome independent of the degree of obesity (Esser et al., 2014). Both clinical data and experimental data from mouse models have demonstrated that liver, muscle, pancreas, and adipose tissue are sites of both ectopic lipid deposition (in non-adipose tissues) (Mittendorfer, 2011; Snel et al., 2012) and inflammation present in metabolic syndrome including obesity, T2D, and non-alcoholic steatohepatitis (NASH) as a result of infiltration of pro-inflammatory macrophages, which secrete pro-inflammatory cytokines such as TNF-α, IL-1β, and IL-6 into these tissues (Chawla et al., 2011; Walenbergh et al., 2013; Esser et al., 2014; Martin-Murphy et al., 2014; McGettigan et al., 2019). During obesity, the adipose tissue in particular has been associated with an accumulation of immune cells in its stromovascular fraction (Chawla et al., 2011) and importantly, increased intra-abdominal visceral fat accumulation is more strongly correlated with metabolic syndrome compared to subcutaneous fat accumulation, suggesting that the pathogenic role of adipose tissue is related to its anatomic location (Koster et al., 2010; Esser et al., 2014). Of critical importance, several clinical studies within the past three decades have demonstrated that obesity and elevated body mass index (BMI) presents a significant risk to lymphatic dysfunction, particularly to female breast cancer patients who are at risk for the development of lymphedema postoperatively (Werner et al., 1991; Meek, 1998; Petrek et al., 2001; Johansson et al., 2002; Meric et al., 2002; Goffman et al., 2004; Ozaslan and Kuru, 2004; Clark et al., 2005; Wilke et al., 2006; McLaughlin et al., 2008; Swenson et al., 2009; Helyer et al., 2010; Vasileiou et al., 2011; Greene et al., 2012; Paskett et al., 2012). Moreover, clinical studies utilizing non-invasive imaging techniques such as near-infrared lymphatic imaging, magnetic resonance imaging, and bioimpedance spectroscopy have characterized lymphatic dysfunction associated with diseases characterized by abnormal adipose tissue accumulation in addition to obesity, such as lipedema and Dercum’s disease (Lohrmann et al., 2009; Rasmussen et al., 2014; Sevick-Muraca et al., 2014; Crescenzi et al., 2018; Crescenzi et al., 2019). Numerous studies within the past two decades using both genetic and diet-manipulated mouse models have now identified key mechanisms underlying the cross-talk of obesity, diabetes, NASH, and metabolic syndrome with lymphatic vascular dysfunction (Table 1), of which several models are discussed in additional detail in this review.

**TABLE 1:** Genetic and diet-induced animal models relating obesity, diabetes, and metabolic syndrome with lymphatic dysfunction.

**Animal model**	**Definition**	**Metabolic and lymphatic phenotypes**	**References**
*apoE*^–/–^ mice	Deficiency of lipoprotein receptor ligand apolipoprotein E	• Delayed lipoprotein clearance • Development of hyper- and dyslipoproteinemia • Development of severe hypercholesterolemia • Prone to development of atherosclerotic lesions • Enlarged initial lymphatic vessels, with decreased lymphatic muscle cell coverage and abnormal distribution of LYVE-1 • Lymphatic leakiness and decreased transport of fluid and dendritic cells • Impaired lymphatic drainage required for reverse cholesterol transport	Lim et al., 2009, 2013; Martel et al., 2013; Lo Sasso et al., 2016
*Ldlr*^–/–^	Deficiency of low-density lipoprotein receptor	• Prone to accelerated atherosclerosis progression with incorporation of a high-fat diet • Pre-treatment with VEGF-C 152S before incorporation of a pro-atherosclerotic diet regimen stimulates lymphangiogenesis of initial vessels, improves lymphatic collecting vessel transport and contractile frequency, and reduces inflammatory cell accumulation	Daugherty et al., 2017; Milasan et al., 2019
*db/db* mice	Mutation resulting in deficiency of the leptin receptor	• Dyslipidemia • Susceptibility to development of obesity-induced type 2 diabetes • Increased lymphatic collecting vessel permeability associated with reduced NO bioavailability • Decreased expression of VEGFR-3 at protein level • Exogenous VEGF-C administration improves wound healing and enhances lymphangiogenesis response • Abnormal lymphatic proliferation in renal cortex and medulla	Kobayashi et al., 2000; Saaristo et al., 2006; Scallan et al., 2015; Wu et al., 2018; Kim et al., 2019
APN-KO mice	Deficiency of adipose-specific plasma adipokine adiponectin, which is down-regulated in association with various obesity-related diseases	• Development of insulin resistance and neointimal formation • Greater susceptibility to microsurgically induced lymphedema associated with reduced lymphatic vascular density	Kubota et al., 2002; Shimizu et al., 2013
Apelin KO mice	Deficiency of apelin ligand, which signals through the G-protein coupled receptor, apelin receptor (APJ)	• Increased susceptibility to weight gain and obesity on high-fat diet • Enlarged lymphatic vessel diameter and increased vessel leakiness in mice on high-fat diet compared to individuals fed normal chow	Sawane et al., 2013
*Prox1*^ + /−^ mice	Haploinsufficiency mutation in the transcription factor Prospero-related homeobox 1 (*Prox1*)	• Subset of mice survive to adulthood but develop adult-onset obesity associated with elevated triglyceride levels • Exhibit abnormal, dilated lymphatics and vascular mispatterning • Characterized by chlyous ascites, chylothorax, and defective lymph transport	Harvey et al., 2005; Escobedo et al., 2016
Chy mutant mice	Heterozygous inactivating mutation in *Vegfr3*	• Characterized by defective lymphatic vessels and appearance of chylous ascites • Display abnormal subcutaneous adipose deposition in the edematous subcutaneous adipose layer • Have increased tissue lipid and collagen content in the skin	Karkkainen et al., 2001; Rutkowski et al., 2010
K14-VEGFR-3-Ig mice	Express a soluble, ligand-binding extracellular portion of VEGFR-3 in the mouse epidermis under the keratin 14 (K14) promoter	• Inhibits dermal lymphatic vessel formation and lymphangiogenesis and exhibits lymphedema phenotype in limbs • As opposed to Chy mice with a similar lack of dermal lymphatics, heterozygous K14-VEGFR-3-Ig mice did not display abnormal collagen or lipid deposition • Protected against high-fat diet-induced obesity and improved systemic insulin sensitivity by suppressing infiltration of pro-inflammatory macrophages via scavenging of VEGF-C and VEGF-D	Makinen et al., 2001; Rutkowski et al., 2010; Karaman et al., 2015
High-fat diet-induced obesity	Increased weight gain and progression to obesity by incorporation of a larger percentage of calories acquired from fats	• Development of insulin resistance, impaired glucose tolerance, dyslipidemia, ectopic lipid accumulation, hepatic steatosis • Decreased lymphatic pumping frequency • Reduction of initial lymphatic vessel density • Increased leakage from lymphatic vessels • Increased perilymphatic inflammation	Weitman et al., 2013; Blum et al., 2014; Savetsky et al., 2014, 2015a; García Nores et al., 2016; Hespe et al., 2016; Nitti et al., 2016
Non-alcoholic steatohepatitis (NASH)	Chronic liver disease and metabolic syndrome characterized by hepatic steatosis, inflammation, and fibrosis. Can be induced by a combination of high-fat diet and oxidized low-density lipoprotein (oxLDL) administration in mice	• NASH-related cirrhosis can result in the development of ascites • Human NASH patients show increased lymphatic vessel density in liver and induction of IL-13 pathway in hepatic LECs • Mice treated with oxLDL show increased hepatic LEC IL-13 production • oxLDL treatment of human LECs reduced *PROX1* expression *in vitro.*	Yimin et al., 2012; Chung and Iwakiri, 2013; Tamburini et al., 2019

Several studies utilizing non-genetic, diet-induced obesity mouse models, in which WT mice have been fed a prolonged high-fat diet compared to control WT mice that are fed normal chow, have demonstrated that increased inflammation induced in obese mice is associated with impaired lymphatic function (Savetsky et al., 2014; García Nores et al., 2016; Hespe et al., 2016; Nitti et al., 2016). Diet-induced obesity in mice was shown to impair lymphatic function as uptake of ^99*m*^Tc-labeled sulfur colloid into sacral lymph nodes after tail injections was decreased in obese mice compared to lean controls and this was exacerbated in obese mice exhibiting lymphedema after surgical tail lymphatic excision. In subcutaneous tissue of the mouse tail, CD4^+^ cell infiltration was significantly increased in obese mice at baseline and the presence of lymphedema in obese mice greatly increased both CD45^+^ and CD4^+^ cell infiltration as well as subcutaneous adipose deposition (Savetsky et al., 2014). Importantly, evidence has shown that impaired lymphatic function and reduced lymphatic density in obese mice is associated with obesity-induced perilymphatic accumulation of inflammatory cells, such as CD3^+^ T cells and CD11b^+^ macrophages, and toxic lipid by-products contributing to the chronic inflammatory environment. Furthermore, comparison of obesity-prone and obesity-resistant mice fed a prolonged high-fat diet demonstrated that dietary changes alone were not sufficient to induce lymphatic dysfunction, thus underscoring the role of obesity-induced inflammation in lymphatic dysfunction (García Nores et al., 2016).

The observation that increased inflammation associated with obesity is correlated with lymphatic dysfunction and reduced vessel density may seem contradictory provided that several studies have shown that under an inflammatory environment, lymphangiogenesis is stimulated as a mechanism for antigen clearance and inflammation resolution in response to pro-lymphangiogenic factors, such as VEGF-C, VEGF-D, and VEGF-A, that are secreted from infiltrating macrophages that respond to chemoattractants expressed by LECs (Kataru et al., 2009; Rahier et al., 2011; Kim et al., 2014). However, other studies have demonstrated that anti-lymphangiogenic cytokines, including IFN-γ and TGF-β1 (Shao and Liu, 2006; Clavin et al., 2008; Oka et al., 2008; Kataru et al., 2011), are elevated in chronic inflammatory responses (Zampell et al., 2012b; Savetsky et al., 2015b; Shin et al., 2015; Kataru et al., 2019) and elevated IFN-γ and TGF-β1 levels were previously detected in obese mice (Winer et al., 2009; Hespe et al., 2016). Additionally, elevated TGF-β1 levels and BMI were observed to have significant partial correlation in human subjects (Yadav et al., 2011). A key observation in studies utilizing both mouse and rat models of obesity and metabolic syndrome is that the lymphatic vessels are leaky and collecting vessel contraction is impaired in subcutaneous and mesenteric collecting vessels (Zawieja et al., 2012; Weitman et al., 2013; Blum et al., 2014; Savetsky et al., 2014, 2015a; García Nores et al., 2016; Hespe et al., 2016; Nitti et al., 2016). While the specific mechanism of lymphatic leakiness in obesity is unclear at this time, it has been proposed that increased perilymphatic accumulation of inflammatory cells, increased production of inflammatory cytokines, and the observation that perilymphatic inducible nitric oxide synthase (iNOS) expression is increased in obese mice may contribute to this phenotype (Hespe et al., 2016). This hypothesis is supported by previous studies demonstrating that inflammatory cytokine signaling enhanced LEC monolayer permeability *in vitro* (Cromer et al., 2014) and increased NO production elevated lymphatic permeability of collecting vessels *in vivo* (Scallan et al., 2015). Furthermore, it was demonstrated that perilymphatic inflammatory cell accumulation may contribute to lymphatic dysfunction and the pathogenesis of obesity via loss of LEC identity as gene expression of *VEGFR-3*, *Prox1*, and *CCL21* was reduced in LECs isolated from sedentary, obese mice compared to lean controls (Hespe et al., 2016). Of note, impaired lymphatic function and leakiness in a subset of *Prox1*^ + /−^ pups results in the effusion of lipid-rich chyle into the abdominal cavity from the mesentery lymphatics and evidence has shown that free fatty acids enriched in lymph promote adipogenesis *in vitro*, thus offering a possible mechanism relating lymphatic dysfunction and adipose tissue accumulation (Escobedo and Oliver, 2017). Support of the existence of cross-talk between the lymphatic system and adipose tissue also comes from recent studies demonstrating that weight loss in mice and reduction of adipose tissue in postmastectomy patients is able to improve lymphatic function and reduce tissue volume accumulation associated with lymphedema (Brorson, 2016; Nitti et al., 2016). Weight loss in a diet-induced obesity mouse model was shown to reverse both perilymphatic inflammation and iNOS expression and improvements were observed in lymphatic vessel density, lymphatic barrier integrity, and collecting vessel contractility (Nitti et al., 2016). Reduction of adipose tissue by longitudinal liposuction treatment with respect to limb was also shown to improve lymphatic function and the reduction of tissue fluid accumulation in patients diagnosed with postmastectomy arm lymphedema (Brorson, 2016) as well as patients diagnosed with primary lymphedema (Schaverien et al., 2018). Together, this data emphasizes the critical role the lymphatic vascular system has as a mediator in the pathogenesis of obesity. As obesity initially contributes to the development of a chronic inflammatory environment, this in turn results in impairment of lymphatic function which can then exacerbate the pathological consequences of obesity and present a significant risk to patients with elevated BMI and the development of lymphedema.

## Impaired Transcription Factor Signaling Related to Lymphatic Dysfunction, Obesity, and Diabetes

The PROX1 transcription factor is critical for LEC fate and differentiation during lymphatic development as well as the formation of the lymphatic vasculature during lymphangiogenesis and maturation into adulthood (Wigle and Oliver, 1999; Wigle et al., 2002; Ma and Oliver, 2017). *Prox1* null mutant mice die between embryonic (E) stage 14.5 and E15.0, and are characterized by severe edema due to the complete absence of the superficial lymphatic vascular network while normal development of the blood vasculature occurs (Wigle and Oliver, 1999). In contrast, *Prox1*^ + /−^ pups also display edema at E14.5 yet maintain formation of a superficial lymphatic vascular plexus. However, many individuals die shortly after birth as a result of severe lymphatic dysfunction and accumulation of chyle in the peritoneal and thoracic cavities (Wigle and Oliver, 1999; Harvey et al., 2005). Surprisingly, a small proportion of *Prox1*^ + /−^ mice generated on the NMRI genetic background are capable of surviving to adulthood (Wigle et al., 1999; Harvey et al., 2005), but develop adult-onset obesity with weight gain noticeable at nine weeks of age (Harvey et al., 2005). These *Prox1*^ + /−^ individuals are characterized by both lymphatic mispatterning in the intestine and mesentery and impaired lymphatic transport as ingested fluorescent lipid leaked from the mesenteric lymphatic collecting vessels, indicative of perturbed vessel integrity and barrier function (Harvey et al., 2005; Escobedo et al., 2016). Therefore, this evidence provides a direct link of lymphatic dysfunction to the development and pathogenesis of obesity. Mechanistically, chyle containing lipid-rich lymph from *Prox1*^ + /−^ mice promoted differentiation of 3T3-L1 preadipocytes into adipocytes. Differentiation was synergistically enhanced by the addition of insulin at a concentration of 10 μg/mL to chyle suggesting the presence of a factor in chyle that cooperates with insulin to promote differentiation. Thus, it was proposed that disruption of lymphatic vascular integrity promotes the ectopic growth of fat in lymphatic-rich regions due to stimulation of preadipocyte differentiation in addition to increased lipid storage in adipocytes (Harvey et al., 2005). Importantly, the development of obesity was directly linked to LEC maintenance of *Prox1* expression in this model as conditional, endothelial cell-*Prox1*^ + /−^ mice similarly were characterized by impaired lymphatic vascular function and the development of obesity (Harvey et al., 2005). Furthermore, restoration of *Prox1* expression specifically within the lymphatic vasculature was capable of rescuing the obese phenotype in *Prox1*^ + /−^ mice (Escobedo et al., 2016).

Several studies in humans have now linked *PROX1* expression to the development of hyperlipidemia, obesity, and T2D (Horra et al., 2009; Kim et al., 2013; Kretowski et al., 2015; Adamska-Patruno et al., 2019). Familial combined hyperlipidemia (FCHL) is a complex genetic dyslipidemia characterized by elevated apolipoprotein B, small, dense LDL particles, triglycerides, and total cholesterol (Ayyobi and Brunzell, 2003). Assessment of *PROX1* expression, as well as the critical lymphatic transcription factor *FOXC2* (Norrmen et al., 2009; Sabine et al., 2012, 2015; Fatima et al., 2016), in subcutaneous adipose tissue of a subset of FCHL patients and healthy control individuals identified a reduction of *PROX1, FOXC2*, or expression of both in the subset of FCHL subjects compared to controls, suggesting that lymphatic dysfunction may contribute to the FCHL phenotype (Horra et al., 2009). Furthermore, a genome-wide association study was performed in families from a Mongolian population that identified the single nucleotide polymorphism (SNP) rs1704198 on chromosome 1q32 associated with larger waist circumference that is located 251 kb away from the *PROX1* gene. Additionally, this SNP was also associated with waist circumference in a replicate study using samples from Korean families and the 1q32 locus was previously reported in a linkage study using an American-Indian diabetes sample (Franceschini et al., 2008; Kim et al., 2013). Recent evidence has identified another SNP in the 5′ UTR of *PROX1* (rs340874) that is a genetic risk factor for T2D (Dupuis et al., 2010; Lecompte et al., 2013; Kretowski et al., 2015; Hamet et al., 2017; Adamska-Patruno et al., 2019). Previous analysis of allelic variants (high-risk CC-genotype carriers and low risk allele T carriers) of the rs340874 SNP demonstrated that carriers of the PROX1 CC genotype presented elevated free fatty acid levels after high-fat meal intake and lower glucose utilization after high-carbohydrate meal intake in comparison to subjections with other PROX1 genotypes. Likewise, carriers of the PROX1 CC genotype were found to have higher visceral fat accumulation despite reduced daily food consumption (Kretowski et al., 2015). Moreover, analysis of non-diabetic PROX1 CC genotype carriers postprandially after challenges with high-carbohydrate (89%) and normo-carbohydrate (45%) meal intake showed altered metabolite profiles compared to low-risk allelic variant carriers including differences in glycerophosphocholine, glycerophosphoethanolamine, sphingolipid, leukotriene, fatty acid, oxidized fatty acid, amino acid, carnitine, bile acids, and amide levels, which may be associated with insulin resistance and T2D development (Adamska-Patruno et al., 2019).

The forkhead-box transcription factor FOXC2 is critical for regulation of lymphangiogenesis during embryonic development (Fatima et al., 2016) and for the formation and maturation of lymphatic valves and collecting vessels postnatally (Norrmen et al., 2009; Sabine et al., 2012, 2015). While inactivating mutations in FOXC2 are predominately associated with the development of a form of hereditary lymphedema, lymphedema-distichiasis syndrome (Fang et al., 2000; Dagenais et al., 2004), previous studies have also linked genetic variation in FOXC2 to obesity and diabetes and identified FOXC2 function as protective against obesity and insulin resistance (Cederberg et al., 2001; Kovacs et al., 2003; Carlsson et al., 2005). In mice, *Foxc2* is found expressed in both white and brown adipose tissue and transgenic mice with increased expression of *Foxc2* in adipose tissue were characterized by reduced cell size in intrabdominal white adipose tissue (Cederberg et al., 2001). Moreover, serum triglyceride, free fatty acids, plasma insulin and glucose content, weight, and lipid content were reduced in *Foxc2* transgenic mice compared to control mice on a high fat diet. Of note, *Foxc2* mRNA levels were also increased in epididymal white adipose tissue of WT mice fed a high-fat diet compared to individuals fed a standard diet, suggesting that *FOXC2* acts as a metabolic regulator in protection against diet-induced obesity and insulin resistance (Cederberg et al., 2001). Clinically, a SNP in the putative *FOXC2* promoter region, a C-512T transition, was identified in a population of Pima Indians which was associated with BMI and percentage of body fat in both male and female subjects as individuals homozygous for the C-512T allele had lower BMI than homozygous C/C or heterozygous C/T individuals. Additionally, the C-512T variant appeared to be involved in female subjects’ regulation of basal glucose turnover and plasma triglyceride levels (Kovacs et al., 2003). Furthermore, an additional study from a Finnish population of T2D patients and control subjects did not identify a significant difference in the C-512T allele and genotype distribution between the study subjects, but the C/C genotype was associated with elevated BMI in T2D (Carlsson et al., 2005). From the patient studies above, it is not known whether the identified mutations are associated with lymphatic dysfunction and in actuality may primarily be related to alterations in hepatocyte function or adipogenic potential. However, the growing evidence supporting the relationship of metabolic syndrome and lymphatic dysfunction warrants further investigation of lymphatic function associated with these described patient mutations. Additionally, individuals suffering from hereditary lymphedema associated with mutations in transcription factors such as *FOXC2* contributing to the development of lymphedema-distichiasis, or *GATA2* contributing to the development Emberger syndrome (Mansour et al., 2010; Ostergaard et al., 2011), may be at risk for abnormal adipose deposition and development of obesity due to proliferation and hypertrophy of adipocytes in lymphedematous tissues (Zampell et al., 2012a; Mehrara and Greene, 2014). Nonetheless, continued studies of these patient populations are required for risk assessment.

## The VEGF-C/VEGF-D/VEGFR-3 Signaling Axis and Lymphatic Dysfunction in the Pathogenesis of Obesity and Diabetes

Vascular endothelial growth factors signaling through their respective VEGF receptor (VEGFR) tyrosine kinases provide a critical driving force for the growth of new vessels during angiogenesis and lymphangiogenesis (Karaman et al., 2018). In the lymphatic vasculature, VEGF-C-induced activation of VEGFR-3 predominately stimulates lymphangiogenesis (Karkkainen et al., 2004). In contrast, VEGF-D, which is capable of activation of both VEGFR-2 and VEGFR-3 in humans (Achen et al., 1998) but only VEGFR-3 in mice (Baldwin et al., 2001), was found to be dispensable for developmental lymphangiogenesis in mice (Baldwin et al., 2005) but its deficiency led to smaller, dysfunctional initial lymphatic vessels in the skin of adult mice (Paquet-Fifield et al., 2013). Independent of its function in the vasculature however, it was recently shown that VEGF-D deficiency significantly elevated cholesterol and triglyceride levels in an atherogenic mouse model via reduction of hepatic expression of syndecan 1, a receptor for chylomicron remnant uptake (Tirronen et al., 2018). In two studies of obese subjects, serum concentrations of VEGF-C were significantly elevated in overweight and obese individuals in addition to VEGF-A (Silha et al., 2005; Gomez-Ambrosi et al., 2010), but elevated VEGF-D was only detected in one report in women after gender segregation (Silha et al., 2005) whereas it was found to be reduced in obese individuals in a separate study using a similar population (Gomez-Ambrosi et al., 2010). It has also been shown in human patients that plasma VEGF-A levels were more strongly correlated with BMI and waist circumference than serum VEGF-C, but VEGF-C levels were more strongly correlated with dyslipidemia (Wada et al., 2011). Importantly, the growth of visceral fat and expansion of white adipose tissue associated with obesity results in a hypoxic environment that activates increased levels of HIF1-α which in turn increases VEGF-A to stimulate angiogenesis and support rapid growth of adipose tissue, but also leads to an upregulation of inflammatory adipokines and promotes tissue fibrosis leading to adipose tissue dysfunction (Rutkowski et al., 2009). Moreover, blocking angiogenesis in young individuals of the obese, leptin deficient *ob/ob* mouse strain via treatment with several inhibitors acting on the endothelium, including TNP-470, angiostatin, and endostatin, prevented expansion of adipose tissue and returned mice to normative metabolic function in adulthood (Rupnick et al., 2002).

More recently, evidence has shown that lymphatic dysfunction associated with inactivation of VEGFR-3, which is classically associated with lymphedema in Milroy’s disease (Butler et al., 2007), is linked to the pathogenesis of obesity and adipose tissue accumulation (Rutkowski et al., 2010) and that targeting this signaling pathway to improve lymphatic function may provide resolution of metabolic syndrome. Two separate mouse models of impaired VEGFR-3 signaling, the Chy (Karkkainen et al., 2001) and K14-VEGFR-3-Ig (Makinen et al., 2001), are both characterized by lack of dermal lymphatic capillaries and presence of lymphedema, but the skin of Chy mice consisted of higher deposition of collagen and fat (Rutkowski et al., 2010). The increase in collagen and lipid content in the tail dermis of Chy mice was attributed to remodeling of collagen and fat density to decrease dermal elasticity and normalize tissue hydraulic conductivity to make tissue easier to swell within the context of edema. Alternatively, fluid accumulation in the K14-VEGFR-3-Ig mice, which expresses a soluble VEGFR-3-Ig construct in the skin under the keratin 14 promoter to trap VEGF-C/D, led to increased hydraulic conductivity to compensate for changes in interstitial fluid pressure, thus highlighting important pathological and functional differences although the lymphedema phenotype is similar in both strains (Rutkowski et al., 2010).

In addition to its role in the lymphatic vasculature, VEGFR-3 is also expressed by a subset of macrophages and VEGF-C and VEGF-D are able to induce their chemotactic migration (Skobe et al., 2001a, b; Schoppmann et al., 2002; Stepanova et al., 2007; Zhang et al., 2014). A more recent study utilized K14-VEGFR-3-Ig mice to investigate the effects of VEGFR-3 signaling inhibition on adipose tissue growth and insulin sensitivity in mice fed a high-fat diet. VEGFR-3 signaling inhibition improved systemic insulin sensitivity and protected against high-fat diet induced fatty liver disease as adipocyte size and adipose tissue inflammation were reduced in K14-VEGFR-3-Ig mice compared to controls (Karaman et al., 2015). In contrast, infiltration of pro-inflammatory M1 macrophages, which express higher levels of *Vegfr3*, was stimulated in control mice fed high-fat diet, which was attributed to elevated adipocyte expression of VEGF-C and VEGF-D. However, blockage of VEGF-C and VEGF-D in K14-VEGFR-3-Ig mice on high-fat diet, or by utilization of a VEGFR-3 blocking antibody in leptin deficient *db/db* mice, protected against the development of insulin resistance and infiltration of pro-inflammatory macrophages. Thus, inhibition of VEGFR-3 signaling suppressed a shift to increased M1/M2 macrophage ratio associated with obesity (Lumeng et al., 2007; Karaman et al., 2015). Furthermore, by utilizing K14-VEGF-C mice that overexpress human VEGF-C in the skin it was shown that these mice more rapidly gained weight, became insulin resistant, had increased ectopic lipid accumulation in adulthood compared to WT littermates on normal chow, and pro-inflammatory macrophage infiltration into adipose tissue was significantly increased (Karaman et al., 2016). Conversely, by stimulating adipose-specific lymphangiogenesis via VEGFR-3 to resolve metabolic syndrome in high-fat diet fed mice, Chakraborty et al. (2019) recently demonstrated that adipose-specific VEGF-D overexpression induced *de novo* lymphangiogenesis and these transgenic mice exhibited improved metabolic responsiveness and reduced obesity-associated macrophage accumulation as immune trafficking was increased from adipose tissue. Furthermore, stimulation of the VEGF-C/VEGF-D/VEGFR-3 signaling axis was shown to improve clearance of contact hypersensitivity-induced inflammation in obese mice as injections of recombinant VEGF-C improved lymphatic function (Savetsky et al., 2015a). Additionally, treatment of atherosclerotic prone, high-fat diet fed *Ldlr^–^^/^^–^* mice with VEGF-C 152S resulted in improved lymphatic molecular transport and reduction of both plaque formation and macrophage accumulation, which was associated predominately with improved lymphatic contraction frequency (Milasan et al., 2019).

Complications from defects in the lymphatic vasculature in diabetic individuals are associated with perturbed wound healing, which can manifest in secondary lymphedema arising from disruption of the lymphatics (Rutkowski et al., 2006; Lin et al., 2013). However, improving VEGFR-3 availability at the cell surface of LECs by inhibition of epsin-mediated degradation was recently shown to improve lymphangiogenesis in diabetic mice, which improved resolution of wound healing and secondary lymphedema in a tail edema model (Wu et al., 2018). Epsins 1 and 2 are ubiquitin-binding adaptor proteins involved in endocytosis and regulate VEGFR-3 bioavailability at the surface of LECs during lymphatic collecting vessel maturation and valve formation (Liu et al., 2014). In the context of adult diabetic mice generated by treatment with streptozotocin and fed a high-fat diet, hyperglycemia-induced oxidative stress increased epsin expression, mediated by phosphorylation of c-Src and the transcription factor AP-1, which promoted degradation of newly synthesized VEGFR-3. Inducible, LEC-specific deletion of epsins was then able to attenuate VEGFR-3 degradation and thus maintained VEGFR-3 bioavailability at the LEC cell surface to improve lymphangiogenesis and restore lymphatic function (Wu et al., 2018). Collectively, these results demonstrate a critical role for the VEGF-C/VEGF-D/VEGFR-3 signaling axis in lymphatic dysfunction associated with the pathogenesis of obesity and metabolic syndrome. Yet from the studies discussed, it is clear that there are underlying contextual factors contributing to the differences observed in Chy and K14-VEGFR-3-Ig models as well as differences in tissue-specific activation of VEGFR-3 signaling. Although both the Chy and K14-VEGFR-3-Ig models are characterized by the lack of dermal lymphatics, the differences in tissue remodeling exhibited by both models in response to changes in interstitial pressure may be attributable to the background strains used, CH3 for Chy mice and C57BL/6 for K14-VEGFR-3-Ig mice, or individual genetic manipulation (Rutkowski et al., 2010). Variations between different mouse strains in immune cell activity, inflammation, vascular remodeling and response, and wound healing have previously been characterized (Bendall et al., 2002; Ryan et al., 2002; Rajnoch et al., 2003; Chan et al., 2005; Marques et al., 2011) as well as the lymphangiogenic response in the mouse cornea micropocket assay and suture-induced corneal neovascularization model (Regenfuss et al., 2010). Furthermore, the differences observed between activation of VEGFR-3 signaling in several of these models is attributable to the physiological responses induced. It was noted that adipose-specific overexpression of VEGF-D resulted in adipose tissue fibrosis, macrophage accumulation, and elevated cytokine levels similar to what was observed in mice with overexpression of VEGF-C under the keratin 14 promoter. However, *de novo* lymphangiogenesis was observed in mice with adipose-specific overexpression of VEGF-D but not mice with overexpressed VEGF-C in the skin (Karaman et al., 2016; Lammoglia et al., 2016; Chakraborty et al., 2019). Therefore, it has been proposed that localization of the initiation of the lymphangiogenic gradient from the adipose tissue, and not the skin, has a role in supporting new lymphatic growth and the resolution of inflammatory cell accumulation in the context of obesity (Chakraborty et al., 2019). Thus, the localization of VEGFR-3 signaling has a critical role in determining physiological responses in the context of obesity and chronic inflammation and the role of adipose-specific VEGFR-3 expression needs to be further elucidated.

While stimulation of lymphangiogenesis is an attractive therapeutic target in obesity and metabolic syndrome to resolve insulin resistance, ectopic lipid deposition, and local immune cell accumulation, careful consideration is needed for the use of therapies stimulating the VEGF-C/VEGF-D/VEGFR-3 signaling axis. Notably, lymphatic vessels become largely independent of VEGF-C/VEGFR-3 signaling after early postnatal development (Karpanen et al., 2006) with the exception of intestinal lacteals, which have been shown to be dependent on VEGF-C/VEGFR-3 signaling for maintenance in adult mice (Nurmi et al., 2015). Furthermore, the effects of tissue-specific activation of VEGFR-3 signaling need to be taken into consideration. As noted previously, there are differences in the lymphangiogenic response between skin-specific and adipose-specific activation of VEGFR-3 (Karaman et al., 2016; Lammoglia et al., 2016; Chakraborty et al., 2019). Additionally, the roles of VEGFR-3 signaling activation beyond the vasculature, such as in regulation of chylomicron remnant removal by hepatocytes and lipoprotein metabolism (Tirronen et al., 2018), need to be carefully considered in the development of therapeutic strategies. Although the development of targeted therapeutics for tissue specific activation of VEGFR-3 is currently a limitation, increasing evidence has shown that targeting this pathway to improve lymphatic function within this pathological context warrants further investigation. Thus, strategies to specifically stimulate adipose-specific lymphangiogenesis may be viable options for therapy to improve inflammatory cell clearance, similar to strategies targeting lymphatic expansion in an inflammatory site-specific manner within the context of a model of chronic skin inflammation (Schwager et al., 2018).

## Abnormal No Signaling and Impaired Lymphatic Function in Obesity and Metabolic Syndrome

Contributing to its pathogenesis, numerous studies utilizing both genetic and non-genetic, diet-induced obesity and metabolic syndrome mouse models (Table 1) have demonstrated that lymph and cholesterol transport, as well as migration of antigen-presenting dendritic cells, is impaired in obese mice compared to lean controls (Lim et al., 2009, 2013; Chakraborty et al., 2010; Dixon, 2010; Martel et al., 2013; Weitman et al., 2013). Underlying this impaired ability of the lymphatic vasculature to support functional transport, several studies have now implicated the role of iNOS and perturbed NO signaling as a mechanism related to poor lymphatic contraction observed in obesity, diabetes, and other models of metabolic syndrome (Zawieja et al., 2012; Blum et al., 2014; Scallan et al., 2015; Hespe et al., 2016; Torrisi et al., 2016). NO is a gaseous free radical that is known to have strong regulatory effects on vasodilation and permeability in the blood vasculature (Kubes and Granger, 1992; Yuan, 2006; Duran et al., 2010) and it is produced by three isoforms of NO synthase, including neuronal NOS (nNOS, NOS I), inducible NOS (iNOS, NOS II), and endothelial NOS (eNOS, NOS III) (Förstermann and Sessa, 2012). In the lymphatic endothelium, eNOS expression, and thus NO production, is regulated by both intracellular calcium levels and vessel shear stress (Leak et al., 1995; Gashev et al., 2002; Tsunemoto et al., 2003). Here, NO signaling serves dual roles as NO produced by LECs, and in particular from the valve/sinus region (Bohlen et al., 2011), functions to control relaxation after a contraction (diastole) in lymphatic collecting vessels (Gashev et al., 2002; Tsunemoto et al., 2003; Gasheva et al., 2006) and exogenous sources of NO have been shown to slow lymphatic contraction frequency (von der Weid et al., 2001; Liao et al., 2011; Zawieja et al., 2016). Thus, NO signaling serves a key role in regulating lymph transport and lymphatic vessel function in a controlled, responsive manner to various stimuli.

As described above, perilymphatic iNOS expression is increased in obese mice associated with an increased inflammatory response (Savetsky et al., 2015a; Hespe et al., 2016; Nitti et al., 2016) and it has been previously shown that under an acute inflammatory response in the mouse, iNOS-expressing inflammatory cells attenuate lymphatic contraction via increased NO production (Liao et al., 2011). Supporting the role of iNOS signaling in the development of impaired lymphatic function and the pathogenesis of obesity, utilization of the selective iNOS inhibitor 1400W was able to improve lymphatic contractile function in obese mice (Torrisi et al., 2016). Moreover, diet-induced weight loss in obese mice also decreased perilymphatic inflammation and iNOS expression, which was also associated with decreased lymphatic leakiness and improved lymphatic contractile function (Nitti et al., 2016). Work from Scallan et al. (2015) has however identified a paradoxical role for NO signaling in the lymphatics in the context of either healthy collecting vessels or those from the *db/db* mouse model of T2D. Here, increased NO production was shown to increase lymphatic permeability in collecting vessels of healthy mice, but restoration of NO signaling in leptin receptor-deficient *db/db* mice was shown to reduce permeability and rescue lymphatic barrier dysfunction. To activate signaling mechanisms functioning downstream of NO signaling in *db/db* mice, the authors demonstrated that pharmacological inhibition of phosphodiesterase 3 (PDE3) was able to rescue lymphatic barrier dysfunction by suppressing PDE3 mediated degradation of cyclic AMP, which activates PKA/Epac1 to maintain endothelial barrier function. It was then proposed that low levels of cGMP from reduced NO bioavailability in diabetic mice relieves the inhibition of PDE3 and in turn, high insulin or leptin levels, as occur in T2D, promote phosphorylation and activation of PDE3 which results in loss of cAMP and impaired signaling through PKA/Epac1 (Scallan et al., 2015). In support of reduced NO bioavailability contributing to an impaired lymphatic function in the context of metabolic syndrome, it was also shown that the thoracic ducts of Sprague-Dawley rats under a high-fructose-fed diet, a model of metabolic syndrome, were characterized by a reduction in eNOS expression coupled to impaired flow responses (Zawieja et al., 2016). *In vitro*, acute insulin signaling activated eNOS phosphorylation mediated by PI3K/Akt signaling in LECs, but prolonged hyperinsulinemia and hyperglycemia promoted insulin resistance, impaired PI3K/Akt/eNOS signaling, mitochondrial function, and NO bioavailability, and also increased lymphatic permeability. The increased lymphatic permeability promoted by hyperglycemia and hyperinsulinemia was then associated with a significant increase in NF-κB nuclear translocation (Lee et al., 2018). While the role of NO signaling within the context of obesity and metabolic syndrome remains incompletely understood, the current data demonstrates that both lymphatic barrier function and contractility are susceptible to elevated levels of NO production, while barrier function is also susceptible to markedly impaired NO production. Evidence from the current studies then suggests that reduction of exogenous NO donors and preservation of endogenous NO synthase function within the lymphatic endothelium may prove as a promising therapeutic strategy.

## Improving Lymphatic Function for Therapeutic Treatment of Metabolic Syndrome

Provided the growing evidence that the relationship between lymphatic dysfunction and the pathogenesis of metabolic syndrome, and obesity in particular, is bi-directional and reciprocating (Mehrara and Greene, 2014; Escobedo and Oliver, 2017), methods to improve lymphatic function have become attractive for therapeutics, including previously proposed VEGF-C related therapies. Several recent studies have proposed other various methods to improve lymphatic function, including both the use of pharmacological agents as well as behavioral and lifestyle changes (Table 2).

**TABLE 2:** Therapeutics targeting lymphatic function improvement for treatment of obesity, diabetes, and metabolic syndrome.

**Therapeutic**	**Target**	**Effect on lymphatic function**	**References**
Tacrolimus	Macrolide calcineurin inhibitor that suppresses T cell proliferation/differentiation	•Reduces perilymphatic inflammation in skin tissue of obese mice •Improves lymphatic transport capacity from distal injection site to the popliteal lymph node •Improves dendritic cell migration capacity and lymphatic contractile function	Torrisi et al., 2016
Cilostamide	Phosphodiesterase 3 (PDE3) inhibitor	•Reduces lymphatic permeability and leakage in collecting vessels of *db/db* mice	Scallan et al., 2015
Y-27632	Rho-associated protein kinase (ROCK) inhibitor	•Induces intestinal lacteal junction “zippering” and conversion of “button-like” cell-cell junctions to linear junctions •Reduces chylomicron uptake into lacteals and transport to mesenteric lymphatics	Zhang et al., 2018
Exercise	Behavioral/lifestyle change with improvements in mediating chronic inflammation, glucose intolerance and endothelial dysfunction in obesity	•Improves lymphatic contractile function •Reduces perilymphatic immune cell accumulation in skin tissue •Reduces expression of inflammatory, anti-lymphangiogenic cytokines including TNF-α, IFN-γ, and IL-1β in skin tissue •Reduces lymphatic leakiness and improves dendritic cell migration capacity •Improves expression of LEC gene expression of *Vegfr3*, *Prox1*, and *Ccl21*	Hespe et al., 2016
Ketogenic diet	Behavioral/lifestyle change associated with incorporation of high-fat, low-carbohydrate diet to improve weight loss	•Increases circulating levels of β—hydroxybutyrate ketone body *in vivo*, which promotes LEC proliferation, migration, and sprouting *in vitro* •Stimulated corneal lymphatic growth after injury •Improved resolution of lymphedema in model of tail microsurgical excision of lymphatic vessels	Keith et al., 2017; García-Caballero et al., 2019

In a model of diet-induced obesity, Torrisi et al. (2016) demonstrated that treatment with topical tacrolimus, an inhibitor of T cell differentiation, improved lymphatic vessel density and lymphatic pumping frequency, reduced perilymphatic accumulation of leukocytes, macrophages, T cells, and perilymphatic iNOS expression in the hindlimbs of mice, whereas direct inhibition of iNOS with the small molecule inhibitor 1400W did not improve lymphatic function. Importantly, adipose tissue volume was not significantly changed at the site of tacrolimus application, suggesting that increased lymphatic density resulted from capillary LEC proliferation with inhibition of obesity-mediated chronic inflammation (Torrisi et al., 2016). In support of targeting perilymphatic inflammation to improve lymphatic function in the context of obesity, it was also shown that behavioral changes to introduce aerobic exercise training, independent of weight loss, were effective in decreasing perilymphatic inflammatory cell accumulation and improving lymphatic function (Hespe et al., 2016). Additional evidence adapting behavioral and lifestyle changes has suggested that long-term incorporation of a high-fat, low-carbohydrate ketogenic diet improves weight loss and limb volume reduction in patients with lymphedema and obesity (Keith et al., 2017). Evidence in mice has demonstrated that LECs rely on fatty acid β-oxidation (FAO) for proliferation, migration, and sprouting, and generation of acetyl-coenzyme A (acetyl-CoA) by FAO facilitates LEC differentiation via acetylation of histone at lymphangiogenic genes, which promotes their expression (Wong et al., 2016). Prolonged incorporation of a high-fat, low-carbohydrate ketogenic diet results in the elevated production of ketone bodies secreted from the liver, which are then able to be oxidized in mitochondria into two molecules of acetyl-CoA in extrahepatic tissues (Puchalska and Crawford, 2017). To characterize the role of ketone body oxidation in the lymphatic vasculature, García-Caballero et al. (2019) recently demonstrated that elevation of lymph ketone bodies in mice, by either a ketogenic diet or administration of the ketone body β-hydroxybutyrate, stimulated lymphangiogenesis after corneal injury and myocardial infarction. Studies investigating high-fat diet induced obesity typically utilize a high-fat diet chow consisting of 60% kcal obtained from fat, 20% from protein, and 20% from carbohydrates resulting in accelerated weight gain and progression to obesity (Weitman et al., 2013; Savetsky et al., 2014; García Nores et al., 2016; Hespe et al., 2016; Nitti et al., 2016; Torrisi et al., 2016). In contrast, high-fat, low-carbohydrate diet (ketogenic) chow generally consists of >70% of kcal obtained from fats, <5% obtained from carbohydrates, and the remaining ∼25% obtained from proteins to induce a ketogenic state and the higher production of ketone bodies as a result of activated FAO (Kennedy et al., 2007; Nilsson et al., 2016; Puchalska and Crawford, 2017). Supporting a stimulated lymphangiogenesis response, ketone body oxidation was shown to increase acetyl-CoA generation, which was incorporated into the TCA cycle to support LEC nucleotide synthesis. Additionally, reducing equivalents generated from oxidation were incorporated for mitochondrial respiration and ATP production. Moreover, it was also shown in a mouse model of secondary lymphedema, via microsurgical ablation of lymphatic vessels in the tail, that a ketogenic diet improved lymphatic function and growth, reduced accumulation of anti-inflammatory CD4^+^ and CD8^+^ T cells, and reduced edema (García-Caballero et al., 2019).

In addition to therapeutically targeting the inflammatory environment to improve lymphatic function, several studies have proposed methods of modifying lymphatic barrier function within the context of obesity and diabetes to improve outcome. As described previously, metabolic syndrome and insulin resistance is associated with impaired NO synthase expression in LECs, resulting in leaky lymphatic collecting vessels and impaired contractile activity. However, Scallan et al. (2015) demonstrated that lymphatic barrier function could be improved in a mouse model of T2D by inhibiting PDE3 with the pharmacological inhibitor cilostamide, thereby providing a method to improve interstitial fluid clearance during inflammation. Additionally, it was also recently proposed that induction of intestinal lacteal junction “zippering” could protect against diet-induced obesity (Zhang et al., 2018). Zhang et al. (2018) recently demonstrated that inducible, pan-endothelial-specific deletion of the VEGF-A receptors VEGFR-1/FLT1 and neuropilin 1 (NRP1) protected mice from high-fat diet-induced obesity. Mice lacking endothelial *Flt1* and *Nrp1* were characterized by lacteals consisting of more linear, “zipper-like” junctions compared to control mice characterized by lacteals with discontinuous “button-like” junctions. This conversion from discontinuous to linear junctions in mutants then prevented absorption of chylomicrons from the intestine. Endothelial-specific deletion of both receptors increased the bioavailability of VEGF-A to activate pathways downstream of LEC VEGFR-2 signaling, which includes inhibition of LEC VE-cadherin cytoskeletal anchoring. Interestingly, short-term treatment of WT mice with the Rho-associated protein kinase (ROCK) inhibitor Y-27632 disrupted cytoskeletal VE-cadherin anchoring in lacteals and induced the formation of linear junctions, mimicking the effect of endothelial-specific deletion of *Flt1* and *Nrp1*, which also resulted in reduced chylomicron absorption (Zhang et al., 2018). Therefore, targeting cytoskeletal regulation of lymphatic junctions may prove promising for therapeutic development to suppress dietary lipid absorption in prevention of and resolution of obesity, but further studies assessing the effects such drugs that regulate cytoskeletal activity are warranted. Collectively, these studies demonstrate that pharmacological or behavioral therapeutics focused on improving lymphatic function may function to improve outcomes in obese and metabolic syndrome patients, including those who also have or are at risk for lymphedema. However, further studies are required to assess how combined therapies may be adapted and to specifically determine what molecular mechanisms underlie perilymphatic inflammatory cell accumulation and lymphatic dysfunction.

## Concluding Remarks

The growing obesity epidemic presents a worldwide health concern as excess weight gain is associated with elevated risk for development of several diseases, but most notably cardiovascular disease and diabetes (Engin, 2017). As the population of obese and metabolic syndrome individuals continues to grow, novel therapeutic developments are required to meet this need. The studies summarized in this review underscore the importance of the lymphatic vascular system in the pathogenesis of obesity, diabetes, and metabolic syndrome, yet our understanding of the molecular mechanisms and signaling pathways involved in the cross-talk between these diseases and the lymphatic system is wholly incomplete. The field of developmental lymphatic biology is continuing to grow as new mechanisms related to LEC differentiation, morphogenesis, maintenance and function continue to be identified, but additional comprehensive studies are required to understand the role of the lymphatic system in pathological states. Moreover, it is important to consider that the lymphatic vasculature interfaces and communicates with numerous tissues, including the blood vasculature, adipose tissue, and inflammatory cells associated with pathological obesity and metabolic syndromes. Therefore, studies investigating combined therapies to target multiple aspects of this pathological environment, such as inhibition of inflammatory cell accumulation, modulation of lymphatic barrier function, and behavioral changes, such as diet modifications and incorporation of exercise regimens, should be of future focus.

## Author Contributions

PN contributed to the writing and editing of the manuscript, and creation of tables. TK contributed to the concepts, editing, and final formatting of the manuscript.

## Conflict of Interest

The authors declare that the research was conducted in the absence of any commercial or financial relationships that could be construed as a potential conflict of interest.
